# Humoral responses to the CoronoVac vaccine in healthcare workers

**DOI:** 10.1590/0037-8682-0209-2022

**Published:** 2023-02-20

**Authors:** Gokhan Eyupoglu, Ramazan Guven, Nuran Karabulut, Adem Cakir, Kemal Sener, Burcu Genc Yavuz, Davut Tekyol, Akkan Avci

**Affiliations:** 1 University of Health Sciences, Basaksehir Cam and Sakura City Hospital, Department of Emergency Medicine, Istanbul, Turkey.; 2 University of Health Sciences, Basaksehir Cam and Sakura City Hospital, Department of Medical Virology, Istanbul, Turkey.; 3 University of Health Sciences, Haydarpasa Numune Training and Research Hospital, Department of Emergency Medicine, Istanbul, Turkey.; 4 University of Health Sciences, Adana City Training and Research Hospital, Department of Emergency Medicine, Adana, Turkey.

**Keywords:** CoronaVac, Healthcare workers, Immunization

## Abstract

**Background:**

This study aimed to assess the immunoglobulin G (IgG) antibody response rate in emergency department (ED) healthcare workers (HCWs) and potential adverse effects after CoronaVac vaccination.

**Methods:**

All included HCWs were grouped based on the previous history of coronavirus disease 2019 (COVID-19) and the number of vaccinations. Furthermore, the IgG antibody response was evaluated based on the sex and smoking status of HCWs. Those with a cut-off index of ≥1.00 after vaccination with CoronaVac were considered to have had COVID-19 and had an adequate humoral response.

**Results:**

Among 224 ED HCWs, 18% experienced the adverse effects of CoronaVac vaccine, the most prevalent being pain in the injection site. The IgG antibody response rate was 20% after the first dose of vaccine, while the response rate increased to 90% after the second dose. Female HCWs had higher IgG response rates compared with male HCWs (53.8 [15.9-147.0] vs 31.2 [4.5-124.0]). Non-smokers had higher IgG response rate compared with smokers (49.0 [11.5-160.5] vs 23.1 [7.4-98.5]).

**Conclusion:**

A single dose of CoronaVac does not produce a sufficient antibody response; hence, two doses are recommended. Men have a lower IgG response compared with women. Smokers had a lower IgG response rate compared with non-smokers. Therefore, it may be necessary to carefully assess the humoral responses of men and smokers when implementing a community vaccination program.

## INTRODUCTION

Numerous countries worldwide are adversely affected by the new waves of coronavirus (COVID-19) pandemic, which began in December 2019 in Wuhan, China^1^. COVID-19 has already infected roughly 177,000,000 individuals worldwide and killed around 3,850,000 individuals^2^. Numerous preventative healthcare measures against COVID-19 have been implemented, including wearing of facemasks and physical distancing, as well as medical treatments using antiviral and anti-inflammatory drugs. However, none of them has been proven effective in controlling the spread of COVID-19^3^. Given the current situation, the most effective method of preventing a pandemic is to create societal immunity via mass vaccination. COVID-19 vaccines are commonly classified into four categories: inactivated viral vaccines, nonreplicating viral vaccines, deoxyribonucleic acid (DNA)-based vaccinations, and messenger ribonucleic acid (mRNA)-based vaccines^4^.

Vaccines against inactivated viruses are made by inactivating virus strains using a variety of physical, chemical, and radioactive methods. Nonreplicating viral vaccines are made using recombinant viral vectors that lack the capacity to replicate within host cells, inducing an immune response without reproducing inside host cells. The adenovirus vaccine, such as the Oxford/AstraZeneca ChAdOx1 new coronavirus (nCoV)-19 vaccine, is the most widely used; meanwhile, DNA-based vaccinations are composed of synthetic DNA fragments carrying the gene encoding the disease-causing protein. Recently discovered mRNA-based vaccines are delivered to the host as a synthetic mRNA strand encoding viral proteins, which generates a powerful immune response against the target protein in the host. mRNA vaccines include Moderna’s mRNA-1273 and Pfizer/BNT162b2 Biontech’s nCoV-19 vaccines^5^.

Typically, the approval and commercial sale of a new vaccine takes several years. On the contrary, the preclinical and clinical trials of vaccine for emergency use in the COVID-19 pandemic were undertaken rapidly and, in some cases, concurrently^6^. A total of 31 vaccines against COVID-19 are in phase 1 trial, 44 vaccines in phase 2 trial, and 30 vaccines in phase 3 trial; fourteen vaccines have been licensed for use after undergoing a phase 3 trial. The World Health Organization (WHO) considered the Pfizer/Biontech (BNT162b2), Janssen (Johnson&Johnson, Ad26.COV2.S), Oxford/AstraZeneca (AZD1222), and Serum Institute of India (Covishield) vaccines to be effective against COVID-19^7^. Additionally, two vaccines have been licensed for use in Turkey: Pfizer/Biontech (BNT162b2) and Sinovac (CoronaVac)^8^.

CoronaVac was developed by a Chinese company. It is Sinovac’s inactivated viral vaccine that has been approved for use in COVID-19 patients in 24 countries to date^7^. The phase 3 trial of CoronaVac was completed in Turkey^9^. It is the first vaccine to be adopted and delivered to healthcare workers (HCWs) with the approval of the Turkish Ministry of Health. Since the first day of the COVID-19 outbreak in the country, the ED HCWs have been in the vanguard of the battle. Hence, this study aimed to ascertain the antibody response in ED HCWs after receiving the CoronaVac vaccine.

## METHODS

The administration of CoronaVac vaccine was initiated on January 14 (2021) in Turkey, and two doses were recommended with a 28-day gap between the first and second doses. The hospital where this study was conducted is one of the vaccination centers and a tertiary referral hospital in Istanbul, Turkey. Approximately 1,500-2,000 patients with suspected COVID-19 visit the emergency pandemic polyclinic of the hospital.

This retrospective study included HCWs who received two doses of CoronaVac, contracted COVID-19 (before 6 months) and were vaccinated with CoronaVac, were infected with COVID-19 but were not vaccinated in the last 6 months, and were not vaccinated despite not having COVID-19. The study participants were divided into five groups based on their COVID-19 history and vaccination status: history of nCoV (−) CoronaVac (−), history of nCoV (+) CoronaVac (−), history of nCoV (−) CoronaVac (one dose), history of nCoV (−) CoronaVac (two doses), and history of nCoV (+) CoronaVac (two doses). Individuals who were infected with COVID-19 or were vaccinated but whose antibody levels were not determined were excluded from this study.

Data on participants’ age, sex, smoking status, post-vaccine side effects, comorbidity, COVID-19 history, and antibody levels were obtained. The antibody level was measured 14 days after the second dose.

The total antibodies against severe acute respiratory syndrome coronavirus 2 (SARS-CoV-2) nucleocapsid protein in serum samples were examined using the Elecsys® anti-SARS-CoV-2 diagnostic kit (Roche Diagnostics, Mannheim, Germany) following the electrochemiluminescence immunoassay method on Cobas e801 instrument (Roche Diagnostics, Mannheim, Germany). Recombinant nucleocapsid antigen was used to detect high-affinity antibodies against SARS-CoV-2 during the Elecsys® anti-SARS-CoV-2 test. The results were interpreted using the cutoff index (COI), which is the value obtained by dividing the detected signal value by the cutoff value. In accordance with the recommendations of the manufacturer, results with a COI of <1 are considered nonreactive and anti-SARS-CoV-2 negative, while those with a COI of ≥1.00 are considered reactive and anti-SARS-CoV-2 positive^10^. The sensitivity and specificity (95% confidence interval) of the kit (reported by the manufacturer) were 99.50% (97-100%) and 99.80% (99.69%-99.88%), respectively^10^.

The study was approved by the Institutional Review Board of Basaksehir Cam and Sakura City Hospital, Istanbul, Turkey (no. 2021-BCSH-1428) and the Advisory Board on Coronavirus Research of the Turkish Ministry of Health. After receiving the ethical approval, the data were retrospectively obtained from the Hospital Automation System and Archives. Due to the retroactive nature of the study, the requirement for obtaining informed consent was waived.

The data obtained in the study were analyzed using the SPSS 24.0 (Armonk, NY: IBM Corp.) software. Categorical variables were expressed as numbers and percentages, while continuous variables were expressed as mean and standard deviation. The distribution of continuous variables was determined using the Shapiro-Wilk test. The Student’s t-test was used for analyzing normally distributed parameters, while the Mann-Whitney U-test was used for between-group comparisons of non-normally distributed parameters. Chi-square or Fisher’s exact test was used to analyze the categorical variables. The significance level was set at 5%.

## RESULTS

In total, 224 ED HCWs participated in this study; among them, 56.3% (n=126) were women with a median (quartiles) age of 25.0 years (24.0-26.0), while 43.8% (n=98) were men with a median (quartiles) age of 27.0 years (25.0-30.0). The demographic characteristics of the study population are shown in Table 1. Approximately 15.3% (n=22) of HCWs vaccinated with CoronaVac developed at least one side effect. Fatigue/arm pain (n=14) and headache (n=5) were the most common side effects. However, none of the patients developed severe side effects that require hospitalization. The HCWs were divided into five groups based on their COVID-19 history and vaccination status:

- History of nCoV (−) CoronaVac (−): n=38
- History of nCoV (+) CoronaVac (−): n=42
- History of nCoV (−) CoronaVac (one dose): n=10
- History of nCoV (−) CoronaVac (two doses): n=103
- History of nCoV (+) CoronaVac (two doses): n=31

**TABLE 1:** Characteristics of the study population.

Age, year	25.5 (24.0-28.0)
Sex, % (n)	
Female	56.3 (126)
Male	43.8 (98)
IgG antibody level, median (quartiles)	26.1 (1.7-98.7)
Comorbid disease, % (n)	
None	95.5 (214)
Asthma	1.3 (3)
Hypothroidism	1.8 (4)
Diabetes mellitus	0.9 (2)
Hypertension	0.4 (1)
Health professionals, % (n)	
Nurse	81.7 (183)
Doctor	18.3 (41)
Smoking, % (n)	
Non-user	65.2 (146)
User	34.8 (78)
Body mass index, % (n)	
<25	49.1 (110)
≥25	50.9 (114)
History of COVID-19, % (n)	
Positive	32.6 (73)
Negative	67.4 (151)
CoronaVac, % (n)	
Non-vaccination	35.7 (80)
Vaccinated with 1 dose	4.5 (10)
Vaccinated with 2 doses	59.8 (134)
Advers events after receiving the CoronaVac vaccine, % (n)	
No vaccine-related advers events	82.0 (118)
Vaccine-related advers events	18.0 (26)
Pain in the injection site	9.7 (14)
Headache	3.4 (5)
Fatigue	3.4 (5)
Heart palpitations	0.7 (1)
Fever	0.7 (1)

**FIGURE 1:**
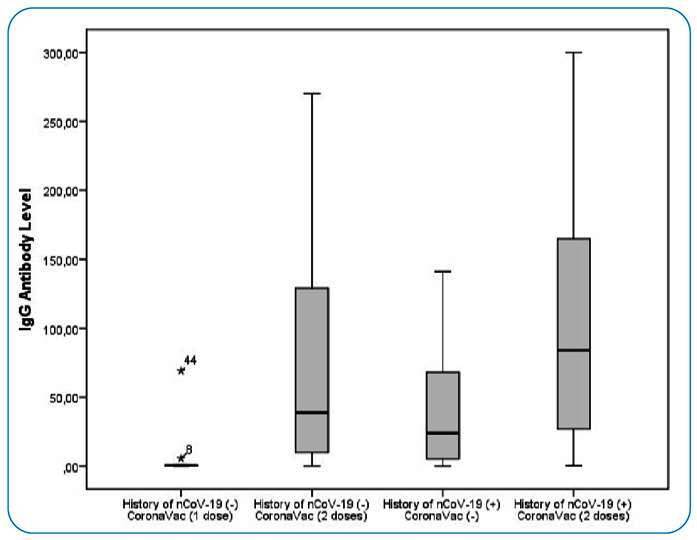
Box and whisker plot of the serum IgG antibody concentrations indicating the median, interquartile range, and 5^th^ and 95^th^ percentiles.

The median (interquartile range) values of immunoglobulin G (IgG) antibody levels based on the vaccination status and nCoV history are shown in Figure 1. The history of nCoV-19 (−) CoronaVac (one dose) group showed inadequate antibody response compared with the history of nCoV-19 (−) CoronaVac (two doses) group (0.6 [0.2-2.1] vs. 39.0 [10.0-131.0]). The IgG antibody (COI) levels of the groups examined based on sex, health profession (nurse/doctor), smoking status, and side effects are shown in Table 2.

**TABLE 2:** IgG antibody levels of emergency department healthcare workers who were divided into various groups according to COVID-19 history and vaccine status, and according to gender, health profession, body mass index, smoking status, and advers events after receiving the CoronaVac vaccine.

	History of nCoV (−)	History of nCoV-19 (+)	History of nCoV-19 (−)	History of nCoV-19 (−)	History of nCoV-19 (+)
	CoronaVac (−)	CoronaVac (−)	CoronaVac (1 dose)	CoronaVac (2 doses)	CoronaVac (2 doses)
	(n=38)	(n=42)	(n=10)	(n=103)	(n=31)
IgG antibody level, COI	0.4 (0.1-0.9)	27.6 (5.3-78.7)	0.6 (0.2-2.1)	39.0 (10.0-131.0)	84.0 (26.0-180.0)
Gender					
Female	0.5 (0.1-4.0)	39.5 (5.4-105.0)	2.9 (0.2-53.1)	53.8 (15.9-147.0)	112.5 (27.4-222.7)
Male	0.3 (0.1-0.6)	13.0 (4.0-70.0)	0.6 (0.1-0.7)	31.2 (4.5-124.0)	70.0 (21.5-119.0)
P value	0.278	0.331	0.454	0.217	0.230
HCW					
Nurse	0.4 (0.1-0.7)	20.3 (4.2-64.2)	0.6 (0.2-2.1)	39.0 (8.9-129.0)	71.0 (20.0-200.0)
Doctor	0.3 (0.1-15.37)	107.0 (39.7-144.5)	-	36.0 (8.7-152.5)	94.5 (58.0-131.2)
P value	0.965	0.035		0.800	0.652
Smoking					
Non-user	0.3 (0.1-1.48)	31.5 (5.8-79.0)	0.6 (0.3-4.3)	49.0 (11.5-160.5)	101.5 (67.5-185.0)
User	0.4 (0.1-14.27)	11.1 (2.5-96.2)	0.4 (0.1-18.0)	23.1 (7.4-98.5)	25.9 (11.5-129.0)
P value	0.750	0.309	0.392	0.304	0.030
AE					
No	-	-	0.6 (0.2-2.1)	39.0 (8.9-126.5)	94.5 (47.5-190.0)
Yes			-	43.0 (16.3-213.0)	17.0 (8.4-119.0)
P value				0.362	0.076

**HCW:** healthcare workers; **BMI:** body mass index; **AE:** advers events after CoronaVac, which did not show a normal distribution (25^th^ vs 75^th^ percentile).

The IgG response (IgG cutoff≥1.0 COI) rates were 90.3% in the history of nCoV-19 (−) CoronaVac (two doses) group and 20.2% in the history of nCoV-19 (−) CoronaVac (one dose) group. The IgG antibody response rate of the groups (p<0.001) are shown in Table 3.

**TABLE 3:** Emergency department healthcare workers classified according to history of COVID-19 and IgG positivity status (antibody level ≥ 1 COI and antibody level < 1 COI).

	IgG antibody negative (<1.00 COI )	IgG antibody positive (≥1.00 COI)	P value
	(n=50)	(n=174)	
History of nCoV (−) CoronaVac (−) % (n)	76.3 (29)	23.7 (9)	
History of nCoV-19 (+) CoronaVac (−) % (n)	4.8 (2)	95.2 (40)	<0.001
History of nCoV-19 (−) CoronaVac (1 dose) % (n)	80.0 (8)	20.0 (2)	
History of nCoV-19 (−) CoronaVac (2 doses) % (n)	9.7 (10)	90.3 (93)	
History of nCoV-19 (+) CoronaVac (2 doses) % (n)	3.2 (1)	96.8 (30)	

## DISCUSSION

The most significant finding of our study on ED HCWs was that a single dose of CoronaVac did not produce an adequate IgG antibody response (20.0%), and the IgG antibody response rate of two doses of CoronaVac was 90.3%. The most common side effects after vaccination were arm pain, headache, and fatigue, which were not fatal and did not require hospitalization.

The CoronaVac developed by the Chinese company Sinovac Biotech against COVID-19 has successfully completed phases 1 and 2 clinical trials. Subsequently, phase 3 trial began in Hong Kong, Chile, Brazil, Indonesia, Turkey, China, and the Philippines^9^. Chile examined the effectiveness of CoronaVac against COVID-19 by dividing the study participants into three groups: unvaccinated, partially vaccinated (one dose), and fully vaccinated (two doses). According to the interim published report, a single dose of CoronaVac prevented COVID-19 by 16% and both doses by 67.0% (reference is needed otherwise it cannot be claimed herein text). In addition, the vaccination also prevented hospitalizations due to COVID-19 in 37.0% of the patients after the administration of a single dose, whereas hospitalization was prevented in 85.0% of the patients after the administration of both doses of CoronaVac. The data regarding the admission of HCWs in intensive care units due to COVID-19 reveal that the administration of the first dose prevented hospitalization in 43.0% of the patients, whereas two doses prevented hospitalization in 89.0% of the patients^11^. The interim/top-line efficacy results (CoronaVac phase 3) of Evidence Assessment of Sinovac/CoronaVac COVID-19 vaccine was published by the WHO in April 2021^12^; it showed that the efficacy rates of CoronaVac against symptomatic cases of COVID-19 were 51% in Brazil, 84% in Turkey, and 65% in Indonesia. Under the phase 4 study conducted in HCWs in Brazil, the vaccine efficacy rate was 50.7% after receiving the second dose^13^. In another phase 4 study conducted in the HCWs in Brazil, the CoronaVac efficacy after a single dose in symptomatic cases of COVID-19 was 49.6%^14^.

Our study showed that the antibody response rate was 20% after the administration of a single dose of CoronaVac. However, after two doses, the vaccine efficacy increased to 90.3%. These results were supported by those of similar interim report (84%) during the CoronaVac phase 3 study conducted in Turkey. A response rate of 50% was reported during the phases 3 and 4 studies in Brazil. In this particular context, the CoronaVac vaccine efficacy was investigated, and the low efficiency was related to the P.1 variant (also known as the Gamma variant), which is quitefatal in young and middle-aged people^15^. In the CoronaVac phase 3 studies conducted in Brazilian, the most common side effects were pain at the injection site, headache, and fatigue^12^.

In the phase 1 and phase 2 studies of the CoronaVac conducted in China, the most common adverse side after the 1st and 2nd doses was pain at the injection site, although no severe side effects occurred in any of the participants^8^. In our study on HCWs, the most common side effects were pain in the injection site, headache, and fatigue, and no severe side effects (requiring hospitalization) were noted in any of the HCWs after vaccination.

In this study, we also analyzed whether the antibody response after contracting COVID-19 is dependent on the sex and smoking status of participants. Results revealed that female HCWs who received both doses of CoronaVac showed a higher IgG antibody response rate compared with the male HCWs. Similarly, non-smokers had a higher antibody response rate compared with smokers.

The effect of smoking on the antibody level in patients with COVID-19 remains unknown; various studies examining the factors affecting immunization have shown that smoking reduces the serum IgG concentration^16^. A previous study was conducted in Italy to evaluate the antibody response according to the gender of the participants after the administration of the BNT162b2 mRNA vaccine to HCWs in Italy^17^. In a review examining the humoral response to the COVID-19 vaccine, the humoral response was lower in smokers^18^. In the study of Ferrara et al., the humoral response of smokers who received two doses of BNT162b2 mRNA vaccine^19^. The participants were divided into various age groups, and the results of the study suggested that female HCWs showed a higher level of antibody response compared with male HCWs in all age groups. In our study, the participants were categorized based on their COVID-19 history and vaccination status (one dose or two doses), and results showed that female HCWs had a higher level of antibody response compared their male counterparts.

The study has some limitations as it was retrospective. The baseline antibody levels of the study population could not be evaluated. In addition, the levels of spike antibodies, neutralizing antibodies, and cellular immunity could not be evaluated. Humoral immunity was examined semiquantitatively in the study. The nucleocapsid antibody kit used in the study is not quantitative, but the COI values measured semiquantitatively may provide an idea about the level of antibody response. Another limitation of this study is that some HCWs had slightly elevated IgG antibody levels, despite the absence of COVID-19 history. Nevertheless, the number of such participants was insignificant (too limited) to influence the study results.

No serious side effects occurred in individuals who were vaccinated, and none of the adverse events due to the CoronaVac vaccine required hospitalization. The IgG response rate was lower in men and smokers.
